# Transcriptome profiling reveals target in primary myelofibrosis together with structural biology study on novel natural inhibitors regarding JAK2

**DOI:** 10.18632/aging.202635

**Published:** 2021-03-03

**Authors:** Weihang Li, Bin Yuan, Yingjing Zhao, Tianxing Lu, Shilei Zhang, Ziyi Ding, Dong Wang, Sheng Zhong, Guangxun Gao, Ming Yan

**Affiliations:** 1 Department of Orthopaedics, Xijing Hospital, The Fourth Military Medical University, Xi’an, China; 2 Department of Hematology, Xijing Hospital, The Fourth Military Medical University, Xi’an, China; 3 Department of Cancer Biology, Dana-Farber Cancer Institute, Boston, MA 02215, USA; 4 College of Clinical Medicine, Jilin University, Changchun, China; 5 Hou Zonglian Medical Experimental Class, Xi'an Jiaotong University, Xi'an, Shaanxi, China; 6 Department of Orthopaedics, Daxing Hospital, Xi’an, China

**Keywords:** bioinformatics, inhibitor, Janus Kinase 2, virtual screening, discovery studio

## Abstract

This study aimed to identify effective targets for carcinogenesis of primary myelofibrosis (PMF), as well as to screen ideal lead compounds with potential inhibition effect on Janus kinase 2 to contribute to the medication design and development.

Gene expression profiles of GSE26049, GSE53482, GSE61629 were obtained from the Gene Expression Omnibus database. The differentially expressed genes were identified, and functional enrichment analyses such as Gene Ontology, protein-protein interaction network etc., were performed step by step. Subsequently, highly-precise computational techniques were conducted to identify potential inhibitors of JAK2. A series of structural biology methods including virtual screening, ADMET (absorption, distribution, metabolism, excretion, and toxicity) prediction, molecule docking, molecular dynamics simulation etc., were implemented to discover novel natural compounds. Results elucidated that PMF patients had abnormal LCN2, JAK2, MMP8, CAMP, DEFA4, LTF, MPO, HBD, STAT4, EBF1 mRNA expression compared to normal patients. Functional enrichment analysis revealed that these genes were mainly enriched in erythrocyte differentiation, neutrophil degranulation and killing cells of other organisms. Two novel natural compounds, ZINC000013513540 and ZINC000004099068 were found binding to JAK2 with favorable interaction energy together with high binding affinity. They were predicted with non-Ames mutagenicity, low-rodent carcinogenicity, less developmental toxicity potential as well as non-toxicity with liver. Molecular dynamics simulation demonstrated that these two complexes: ZINC000013513540-JAK2 and ZINC000004099068-JAK2 could exist stably under natural circumstances. In conclusion, this study revealed hub genes in the carcinogenesis of PMF. ZINC000013513540 and ZINC000004099068 were promising drugs in dealing with PMF. This study may also accelerate exploration of new drugs.

## INTRODUCTION

Primary Myelofibrosis (PMF), one of the myeloproliferative neoplasms (MPNs) which arises from clonal proliferation of hematopoietic stem cells and leads to progressive bone marrow (BM) fibrosis, is currently classified with polycythemia vera (PV) and essential thrombocythemia (ET) under the broad WHO category of MPNs [1]. The main pathological manifestations are marked reactive bone marrow fibrosis, osteosclerosis, angiogenesis, extramedullary hematopoiesis (EMH) and abnormal cytokine expression. Patients with PMF frequently complain about fatigue as well as symptoms due to splenomegaly, such as ascites, fever and night sweats [2]. Besides, constitutional symptoms including severe anemia, marked hepatosplenomegaly, bone pain, infarct, pruritus, cachexia, thrombosis and bleeding were also reported appearing in PMF [3–5].

The overall prognosis of patients with PMF is generally poor, with a short median survival and poor life quality. The estimated median survival time was 15 years for patients younger than 60, and 6 years for patients older than 60 [5, 6]. Poor life quality may due to constitutional symptoms and cachexia. Unfortunately, current chemotherapy of PMF including JAK2 inhibitors do not provide a promising prospect view [7, 8]. Fedratinib (TG101348) is a JAK2-selective inhibitor which demonstrated clinical benefits in patients with MF in early-phase clinical trials, and is approved by the Food and Drug Administration (FDA) as the first drug to treat myelofibrosis [9]. Currently, only this drug has shown a relatively efficient efficacy, the disadvantage of this inhibitor also remains obvious and needs to be improved. Therefore, there is an urgent need to discover more innovative drug candidates regarding PMF in order to improve this situation.

In recent years, with the development of bioinformatics and microarray technology, they were widely employed to analyze malignant neoplasms. It helps us to study initiation, progression and metastasis of carcinoma under molecular level, making it possible to analyze the genetic alteration and molecular mechanisms in the development of PMF. Bioinformatics analysis allows researchers reveal molecular therapeutic target and provide a theory basis through a systematic, effective and accurate manner. Natural compounds and their derivatives always provide unique chemical structures as well as potential biological functions in today’s pharmacologic market, due to their malleable and readily available property, they have made a great contribution to medication screening [10, 11]. Small molecules and natural products screening are an essential aspect if not the first means to tackle an emergent or uncontrollable disease, these molecules and approaches have been used to improve chemotherapy as well as overall cancer treatment [12, 13]. Consequently, bioinformatics combined with structural biology were implemented in this study to accelerate the discovery of PMF drugs.

**Figure 1:**
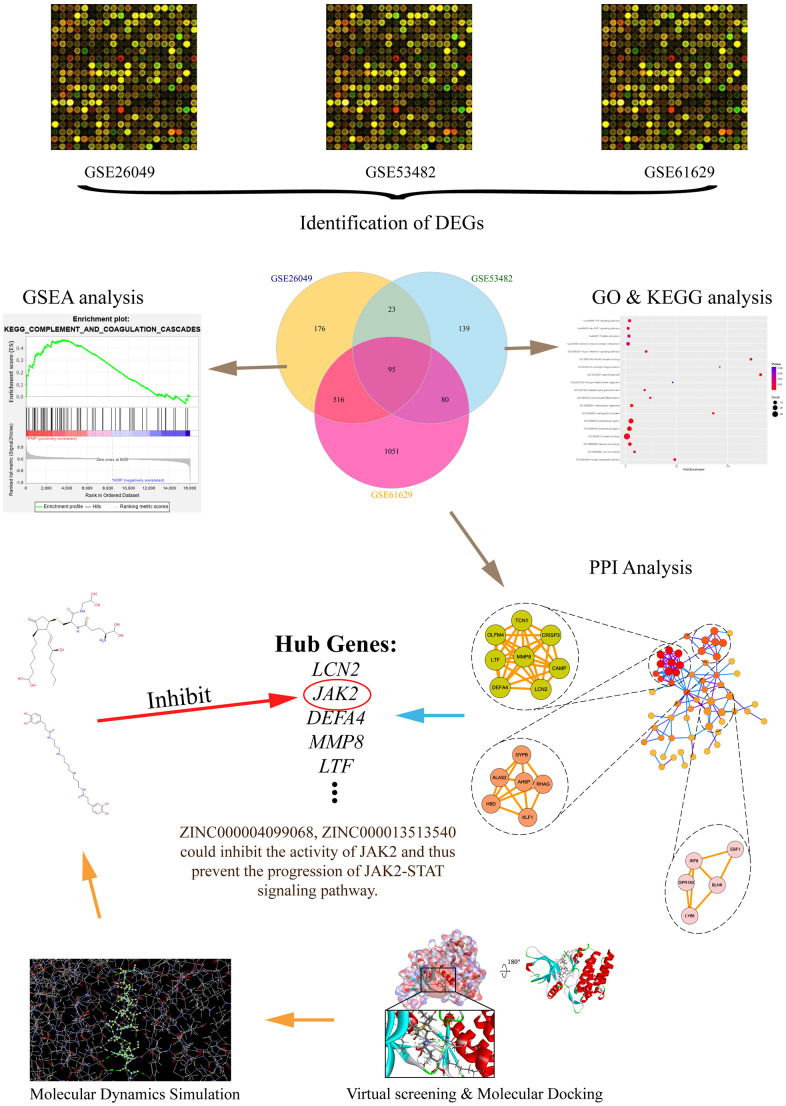
**The whole diagram and framework of this study.** DEGs, differentially expressed genes; GO, Gene Otology; KEGG, Kyoto Encyclopedia of Genes and Genomes; GSEA, Gene set enrichment analysis; PPI, protein-protein interaction.

In the aspect of MPN, it is well known that the accurate differential diagnosis is the key to conducting prognosis as well as therapy [14]. Firstly, this study aimed at identifying hub genes through bioinformatics method in the occurrence of PMF. Totally 3 messenger RNA microarray datasets (GSE26049, GSE53482, GSE61629) involving primary myelofibrosis were downloaded from the Gene Expression Omnibus (GEO) to identify driver genes and key pathways causing the progression of PMF. Subsequently, Gene Ontology (GO) and Kyoto Encyclopedia of Genes and Genomes (KEGG) analyses were performed to discover molecular function changes and abnormal signaling pathways by showing their biological process, cellular component and molecular function. Protein-protein interaction network analysis (PPI) was then carried out to visualize the connection between different genes. After identifying the hub genes causing the occurrence of PMF, this study further employed a series of structural biological and chemical methods (such as virtual screening, molecular docking, toxicity prediction, molecular dynamics simulation etc.) to screen and identify novel lead compounds that could have biological effects targeting hub genes in the treatment of PMF. This may contribute to new ideas and resources for drug discovery in the pharmaceutical market. This study provided a list of drug candidates as well as their pharmacologic properties from ZINC15 database, which could offer a solid practical foundation for gene product inhibitors’ research. The whole diagram and framework of this study were shown in Figure 1.

## RESULTS

### Expression profiles’ quality control and samples selection

**Figure 2:**
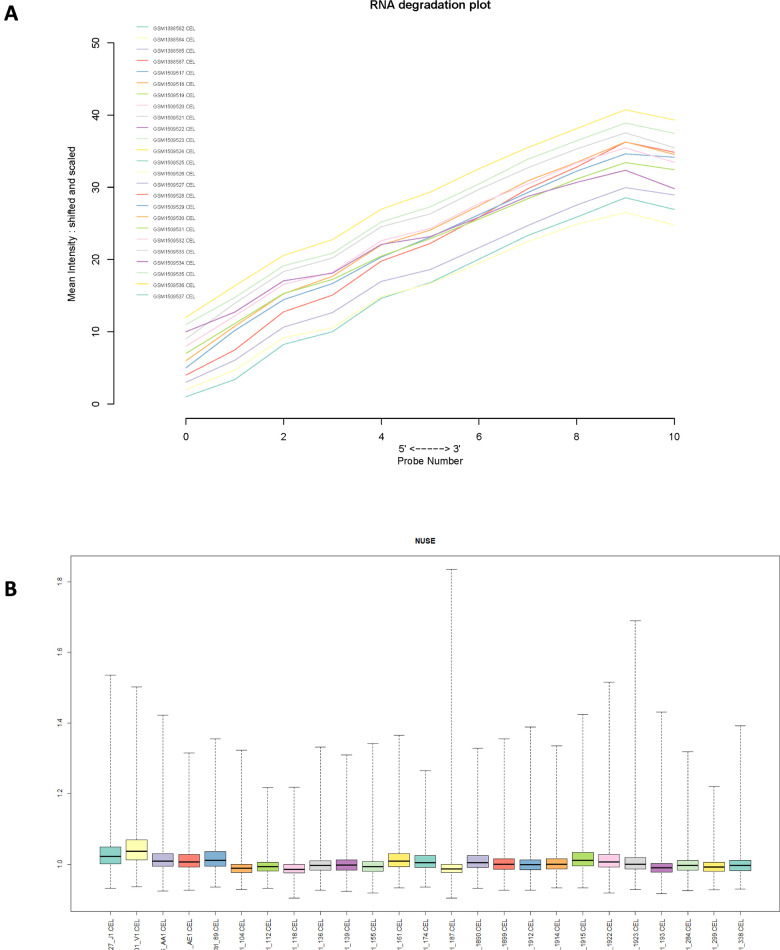
(**A**) RNA degradation plot of GSE61629 and (**B**) Box plot of normalized unscaled standard errors (NUSE) of GSE61629, which were used for quality control.

Normalized unscaled standard errors (NUSE) of each GSE series were calculated in this study, then box plots of gene expression values were plotted for each sample. Next, RNA degradation curve, box plot and gray scale image of each sample were plotted in each GSE series (Figure 2 and Supplementary Figure 1). Totally, 2 samples were removed due to their poor chip quality. As a result, 54 PMF samples and 72 normal samples were selected as standard data for further analysis.

### Eliminating batch effects in each GSE series

**Figure 3:**
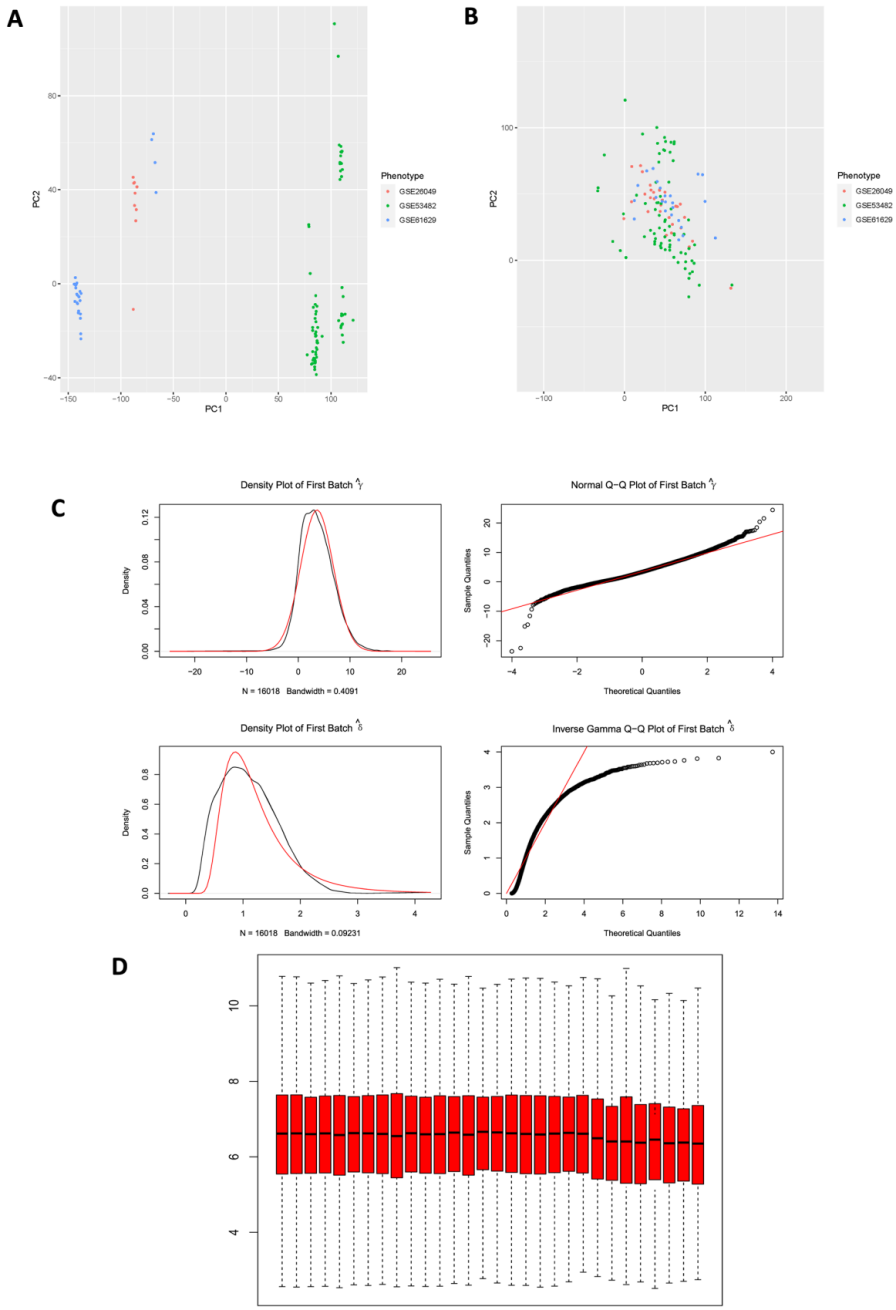
(**A**) PCA plot among 3 datasets before eliminating “batch effects”. PCA, principal component analysis. (**B**) PCA plot among 3 datasets after eliminating “batch effects”. (**C**) Generated QQ plot and density plot after eliminating “batch effects”. (**D**) Validation of box plot of GSE26049 after eliminating “batch effects”, results illustrated that the median of each sample expression was on a straight line, indicating that the operation of removing “batch effects” did not affect the expression of each dataset.

Microarray experiments are costly and time-consuming, many of the studies use multiple arrays, at different times, on different array chargers or even on different microarray platforms. Here, terminology “batch” refers to microarray processed at one site over a short period of time using the same platform, while the cumulative error introduced by time-dependent and place-dependent experimental variations is called “batch effects” [15, 16], which could influence the results of different microarray experiments and thus mask or confound real biological differences. Principal Component Analysis (PCA) was firstly conducted to reduce dimension for these 3 GSE series in order to determine whether “batch effects” existed among them (Figure 3A), results illustrated that there was a significant difference among these 3 GSE series. Subsequently, “combat” function (“sva” package in R) was applied to eliminate “batch effects” in these series, and then PCA was performed again to validate the results (Figure 3B), results displayed non-difference in point distribution on PC1 axis. In addition, QQ plot and density plot generated by eliminating “batch effects” were shown (Figure 3C) to visualize and validate the results. Box plot of GSE26049 showed that the median of each sample’s expression quantity was on a straight line, indicating that removing “batch effects” did not affect the expression pattern of each dataset (Figure 3D).

### Identification of DEGs in PMF

**Figure 4:**
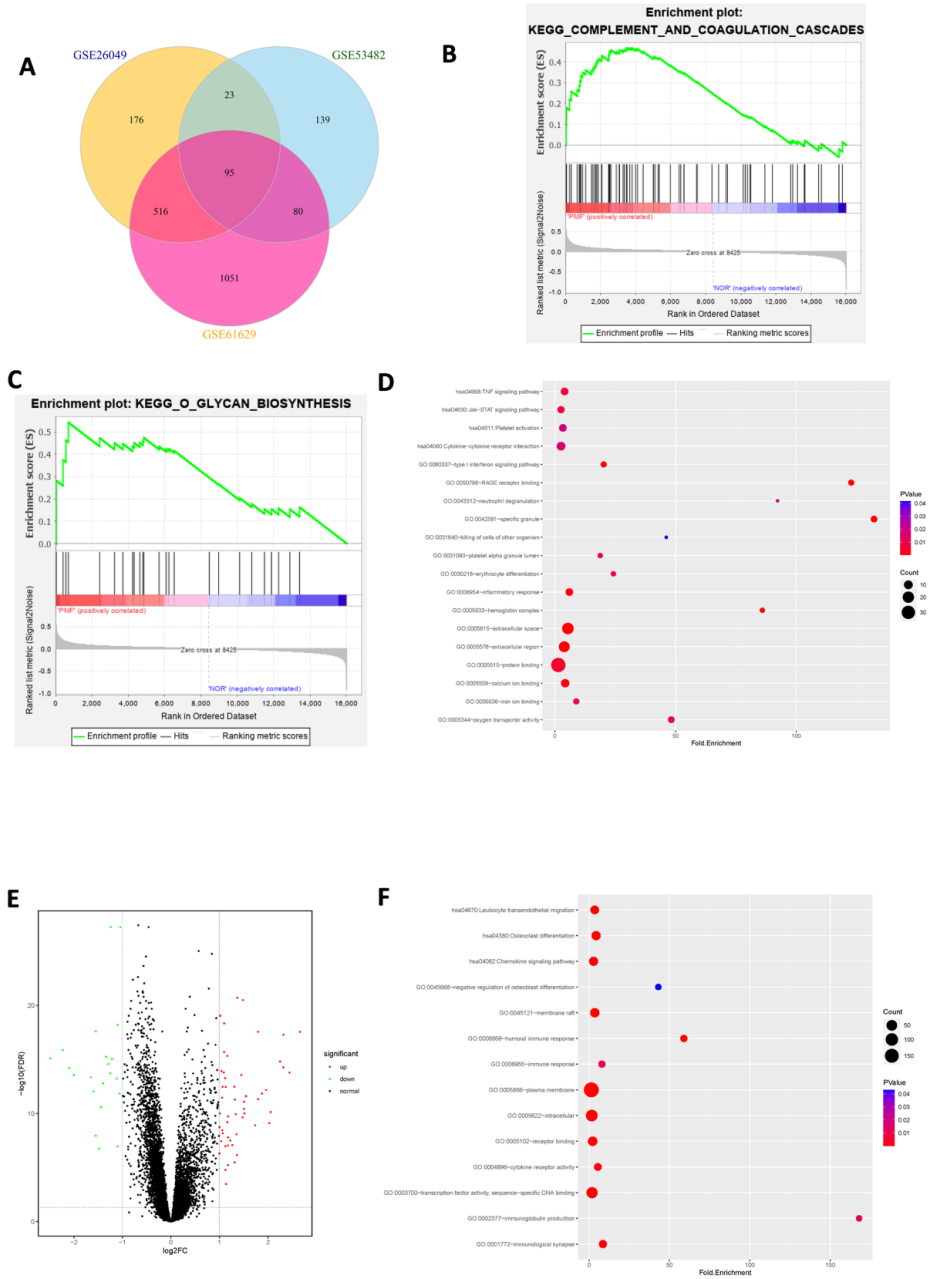
(**A**) Venn plot of differentially expressed genes among 3 datasets. (**B**) Gene set enrichment analysis of mutual DEGs among 3 datasets. (**C**) Gene set enrichment analysis of mutual DEGs among 3 datasets. (**D**) Bubble chart of functional and pathway enrichment analysis of up-regulated genes. (**E**) Volcano plot of differentially expressed genes among 3 datasets. Red represented up-regulated genes, green represented down-regulated genes and black represented normal genes. (**F**) Bubble chart of functional and pathway enrichment analysis of down-regulated genes.

After standardization of the microarray data, we analyzed the gene expression profiles of these datasets by comparing PMF samples and normal samples to identify DEGs in GSE26049, GSE53482 and GSE61629. In summary, 810 DEGs were screened from GSE26049, of which 143 up-regulated genes and 667 down-regulated genes. A total of 337 DEGs were identified from GSE53482, among which 308 genes were up-regulated and 29 genes were down-regulated. And there was 1742 DEGs in GSE61629, among which 1560 genes were up-regulated, and 182 genes were down-regulated. Altogether, 95 mutual DEGs among these 3 datasets were integrated by conducting Venn plot analysis (Figure 4A).

### Functional and pathway enrichment analysis of PMF

The integrated up-regulated and down-regulated DEGs were uploaded into DAVID database for further investigation of functions and signaling pathways. The detailed results of GO and KEGG pathway analyses as well as GSEA analysis were shown in Table 1 and Figure 4B–4F. GO analysis revealed that mutual up-regulated DEGs were mainly associated with several biological processes (BPs, including erythrocyte differentiation, neutrophil degranulation, killing of cells of other organism etc.); molecular functions (MFs, including protein binding, iron ion binding, oxygen transporter activity etc.) and cellular components (CCs, including extracellular region, specific granule, hemoglobin complex etc.), while down-regulated DEGs were significantly enriched in humoral immune response, transcription factor binding and plasma membrane. KEGG analysis indicated that mutual up-regulated DEGs were chiefly involved in cell cycle, JAK-STAT signaling pathway, TNF signaling pathway whereas down-regulated DEGs were mostly associated with osteoclast differentiation etc. In addition, Gene Set Enrichment Analysis (GSEA) suggested that mutual DEGs were primarily enriched in complement and coagulation cascades, O-glycan biosynthesis etc.

**Table 1:** Functional and pathway enrichment analysis of up-regulated and down-regulated DEGs among 3 GSE datasets.

**Expression**	**Category**	**Term**	**Count**	**%**	**P-value**
Upregulated	GOTERM_BP_DIRECT	GO:0031640~killing of cells of other organism	2	3.703704	0.0041707
	GOTERM_BP_DIRECT	GO:0043312~neutrophil degranulation	2	3.703704	0.021071
	GOTERM_BP_DIRECT	GO:0030218~erythrocyte differentiation	3	5.555556	0.006553
	GOTERM_BP_DIRECT	GO:0060337~type I interferon signaling pathway	4	7.407407	9.65E-04
	GOTERM_BP_DIRECT	GO:0006954~inflammatory response	7	12.96296	9.71E-04
	GOTERM_CC_DIRECT	GO:0005576~extracellular region	18	33.33333	1.22E-06
	GOTERM_CC_DIRECT	GO:0042581~specific granule	5	9.259259	4.13E-08
	GOTERM_CC_DIRECT	GO:0005833~hemoglobin complex	3	5.555556	5.17E-04
	GOTERM_CC_DIRECT	GO:0005615~extracellular space	21	38.88889	2.63E-10
	GOTERM_CC_DIRECT	GO:0031093~platelet alpha granule lumen	3	5.555556	0.010767
	GOTERM_MF_DIRECT	GO:0050786~RAGE receptor binding	4	7.407407	3.73E-06
	GOTERM_MF_DIRECT	GO:0005515~protein binding	36	66.66667	0.00446
	GOTERM_MF_DIRECT	GO:0005506~iron ion binding	4	7.407407	0.009918
	GOTERM_MF_DIRECT	GO:0005509~calcium ion binding	9	16.66667	9.81E-04
	GOTERM_MF_DIRECT	GO:0005344~oxygen transporter activity	5	3.703704	0.009918
	KEGG_PATHWAY	hsa04630:Jak-STAT signaling pathway	6	1.980198	0.007224
	KEGG_PATHWAY	hsa04060:Cytokine-cytokine receptor interaction	10	3.30033	0.007196
	KEGG_PATHWAY	hsa04668:TNF signaling pathway	7	2.310231	0.007773
	KEGG_PATHWAY	hsa04611:Platelet activation	7	2.310231	0.001902
Downregulated	GOTERM_BP_DIRECT	GO:0006959~humoral immune response	4	20	3.46E-05
	GOTERM_BP_DIRECT	GO:0002377~immunoglobulin production	2	10	0.001126
	GOTERM_CC_DIRECT	GO:0005886~plasma membrane	194	29.61832	6.60E-07
	GOTERM_CC_DIRECT	GO:0001772~immunological synapse	10	1.526718	1.49E-06
	GOTERM_CC_DIRECT	GO:0005622~intracellular	76	11.60305	1.31E-05
	GOTERM_MF_DIRECT	GO:0005102~receptor binding	28	4.274809	1.73E-04
	GOTERM_MF_DIRECT	GO:0004896~cytokine receptor activity	7	1.068702	0.001569
	KEGG_PATHWAY	hsa04062:Chemokine signaling pathway	22	3.358779	2.93E-05
	KEGG_PATHWAY	hsa04380:Osteoclast differentiation	24	3.664122	3.50E-09
	KEGG_PATHWAY	hsa04670:Leukocyte transendothelial migration	17	2.59542	2.07E-05

### Module screening and hub genes selection from the PPI network

**Figure 5:**
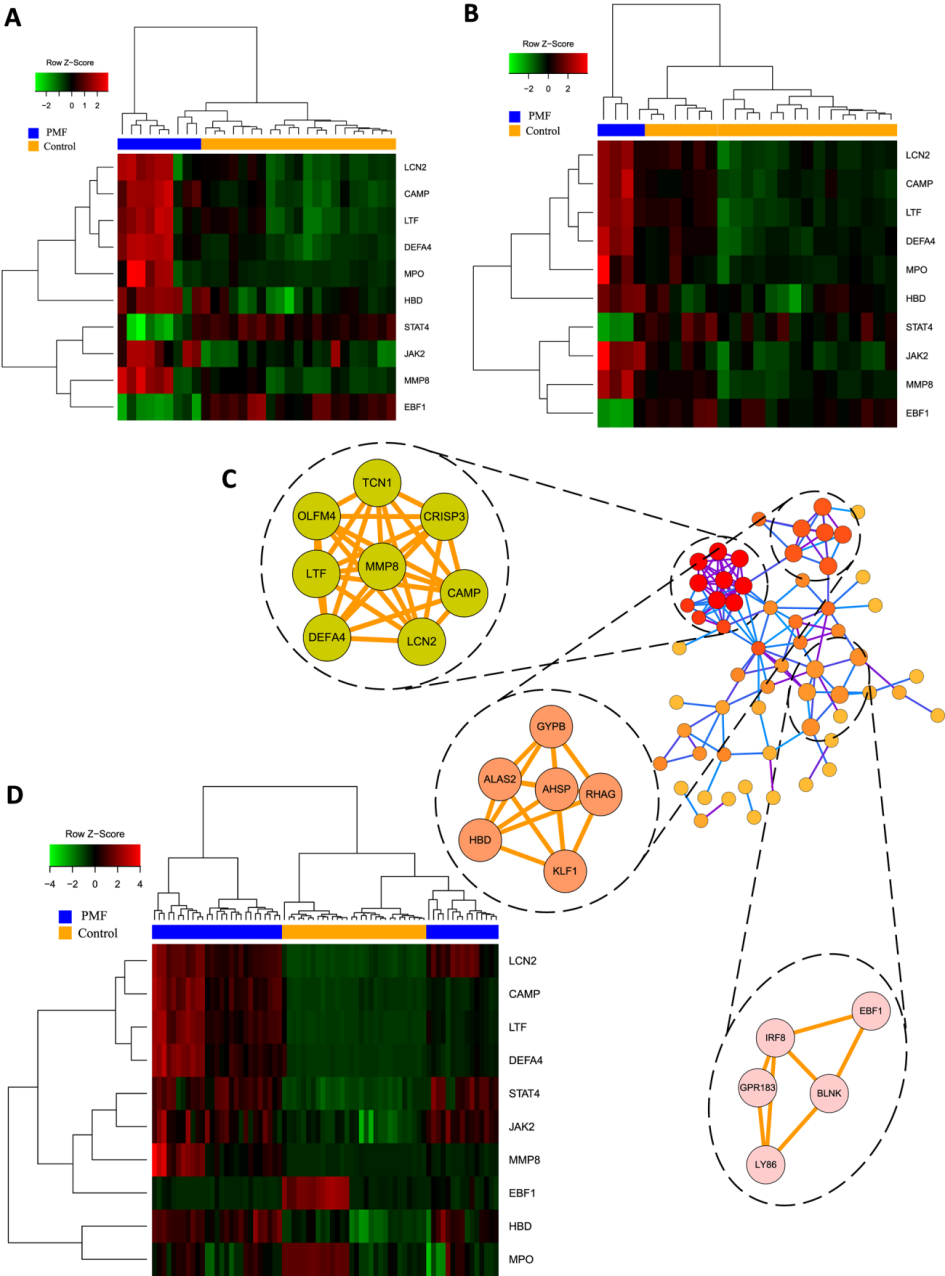
(**A**) Hub genes expression heatmap of GSE26049. (**B**) Hub genes expression heatmap of GSE61629. (**C**) Visualization of protein-protein interaction network and the top 3 modules from the PPI network. (**D**) Hub genes expression heatmap of GSE53482.

The former 95 mutual DEGs among these 3 datasets were constructed with PPI network and statistically significant modules were obtained through Cytoscape 3.7.0. After MCODE analysis, 60 nodes and 132 edges were generated, together with the top 3 significant modules, as shown in Figure 5C. The functional annotation and pathway enrichment of these modules were also conducted on DAVID database, as shown in Table 2. Results revealed that genes in module 1 chiefly functioned in the innate immune response, extracellular region whereas in modules 2 and 3, genes were mainly enriched in erythrocyte differentiation, hemoglobin complex and humoral immune response.

**Table 2:** Functional and pathway enrichment analysis of the module genes.

**Module**	**Term**	**Count**	**%**	**P-value**
1	GO:0019731~antibacterial humoral response	3	37.5	1.40E-04
	GO:0045087~innate immune response	4	50	5.41E-04
	GO:0005576~extracellular region	7	87.5	3.05E-06
	GO:0005506~iron ion binding	2	25	0.035767
2	GO:0030218~erythrocyte differentiation	3	42.85714	3.31E-05
	GO:0005833~hemoglobin complex	2	28.57143	0.003288
3	GO:0006959~humoral immune response	3	60	6.76E-05

Hub genes were identified using “cytohubba” plug-in which contained 11 different topological analyses for researchers to find out the most linked genes across complex networks. This study screened hub genes through calculating nodes’ scores among different genes and ranking them. Eventually the mutual hub genes were identified by intersecting the top 25 genes of each algorithm. Altogether, 10 genes were recognized as hub genes, including LCN2, JAK2, MMP8, CAMP, DEFA4, LTF, MPO, HBD, STAT4, EBF1, as listed in Table 3. Hierarchical clustering analysis demonstrated that the hub genes could significantly differentiate the PMF samples from the normal samples (Figure 5A, 5B, 5D). Among them, JAK2 and LCN2 ranked highest in these 11 algorithms.

**Table 3:** Detailed information of 11 topological analysis methods of hub genes.

**Gene symbol**	**MCC**	**DMNC**	**MNC**	**Degree**	**EPC**	**Bottleneck**	**EcCentricity**	**Closeness**	**Radiality**	**Betweenness**	**Stress**
LCN2	5424	0.61085	11	11	23.632	2	0.2	25.28333	5.58824	102.5119	440
JAK2	5280	0.61702	11	11	23.153	2	0.2	24.2124	5.42210	138.2541	350
MMP8	5286	0.61853	10	10	23.533	1	0.2	23.11667	5.23529	38.82381	140
CAMP	5437	0.57076	12	13	23.792	9	0.2	27.86667	5.84314	547.2548	1094
DEFA4	5424	0.61085	11	11	23.737	6	0.2	25.28333	5.58824	102.5119	440
LTF	5280	0.69213	9	9	23.313	1	0.16667	21.7	5.03922	4.3	20
MPO	397	0.57279	9	10	23.562	3	0.2	25.28333	5.64706	167.3024	458
HBD	55	0.52304	6	7	14.675	8	0.2	22.28333	5.2549	361.7238	484
STAT4	9	0.37893	4	5	18.797	2	0.25	22.91667	5.58824	127.9548	330
EBF1	5	0.30898	3	4	14.844	3	0.2	20.4	5.21569	219.6071	484

### Virtual screening of natural products regarding JAK2

The ligand binding pocket is a pivotal regulatory site of JAK2, as small molecules binding to this region could inhibit the activity of JAK2 and thus prevent the functions and its downstream signaling pathways (e.g., JAK2-STAT signaling pathway). Therefore, the pocket region was selected as docking site. Altogether, 17799 biogenic-for sale-named natural product compounds were downloaded from ZINC15 database. Crystal structure of JAK2 (PDB ID: 4JI9) was selected as the receptor protein and Fedratinib (TG101348, PDB ID: 4PS5) was chosen as reference ligand to compare the pharmacologic properties with other compounds. After virtual screening, 10102 compounds were found binding with JAK2 stably by libdock module. Among those, 667 compounds had higher libdock score than the reference ligand Fedratinib (libdock score:129.056). The top 20 ranked compounds and Fedratinib based on libdock score were listed in Table 4.

**Table 4:** Top 20 ranked compounds with higher libdock scores than Fedratinib and the reference ligand score.

**Number**	**Compounds**	**Libdock score**
1	ZINC000085545908	205.324
2	ZINC000062238222	200.453
3	ZINC000095620524	194.484
4	ZINC000096015174	185.064
5	ZINC000004099069	184.678
6	ZINC000004096684	183.854
7	ZINC000085544839	182.698
8	ZINC000085826837	182.089
9	ZINC000013513540	182.074
10	ZINC000072131515	181.814
11	ZINC000004099068	180.848
12	ZINC000008552069	180.649
13	ZINC000011616635	179.398
14	ZINC000014951634	178.563
15	ZINC000150338786	177.925
16	ZINC000042805482	177.525
17	ZINC000004096878	177.273
18	ZINC000004654845	176.016
19	ZINC000056897657	175.962
20	ZINC000014712793	175.38
21	Fedratinib (reference ligand)	129.056

### Pharmacologic properties predictions of compounds

Pharmacologic properties of all the identified compounds as well as Fedratinib were firstly predicted with ADME module of DS4.5, including aqueous solubility, CYP2D6 inhibition, hepatotoxicity, blood-brain-barrier level, human intestinal absorption, and plasm protein binding properties. As shown in Table 5, the aqueous solubility level (defined in water at 25° C) illustrated that 10 compounds had good solubility in water (defined as score ≥ 3), which were better than Fedratinib (solubility level: 1); all compounds but ZINC000004654845 were predicted with non-inhibition with CYP2D6, an essential enzyme in drug metabolism. For hepatotoxicity, 13 compounds were predicted with non-hepatotoxicity regarding liver whereas Fedratinib was hepatotoxic drug. All compounds were discovered to be high-permeability with blood-brain-barrier. For human intestinal absorption, totally 19 compounds had better intestinal absorption level (score: 3) than Fedratinib did (score: 2). Finally, plasma protein binding properties indicated that 5 compounds had the same strong binding force as Fedratinib did.

**Table 5:** ADME (Absorption, Distribution, Metabolism and Excretion) properties of the candidate compounds.

**Number**	**Compounds**	**Solubility level**^1^	**BBB level**^2^	**CYP2D6**^3^	**Hepatotoxicity**^4^	**Absorption level**^5^	**PPB level**^6^
1	ZINC000013513540	4	4	0	0	3	0
2	ZINC000095620524	4	4	0	1	3	0
3	ZINC000056897657	1	4	0	1	3	0
4	ZINC000014951634	3	4	0	0	3	0
5	ZINC000011616635	2	4	0	0	3	0
6	ZINC000004096878	1	4	0	1	3	1
7	ZINC000014712793	4	4	0	0	3	0
8	ZINC000150338786	1	4	0	1	3	1
9	ZINC000085545908	3	4	0	0	3	0
10	ZINC000085544839	3	4	0	1	3	0
11	ZINC000062238222	3	4	0	1	3	0
12	ZINC000008552069	4	4	0	1	3	0
13	ZINC000004096684	1	4	0	0	3	1
14	ZINC000004099068	3	4	0	0	3	0
15	ZINC000004099069	3	4	0	0	3	0
16	ZINC000004654845	1	4	1	0	3	1
17	ZINC000096015174	1	4	0	0	3	0
18	ZINC000072131515	0	4	0	0	3	1
19	ZINC000085826837	2	4	0	0	2	0
20	ZINC000042805482	2	4	0	0	2	0
21	Fedratinib (reference)	1	4	0	1	2	1

^1^Aqueous-solubility level: 0 (extremely low); 1 (very low, but possible); 2 (low); 3 (good);

^2^Blood Brain Barrier level: 0 (Very high penetrant); 1 (High); 2 (Medium); 3 (Low); 4 (Undefined);

^3^Cytochrome P450 2D6 inhibition: 0 (Non-inhibitor); 1 (Inhibitor);

^4^Hepatotoxicity: 0 (Nontoxic); 1 (Toxic);

^5^Human-intestinal absorption level: 0 (good); 1 (moderate); 2 (poor); 3 (very poor);

^6^Plasma Protein Binding: 0 (Absorbent weak); 1 (Absorbent strong).

**Figure 6:**
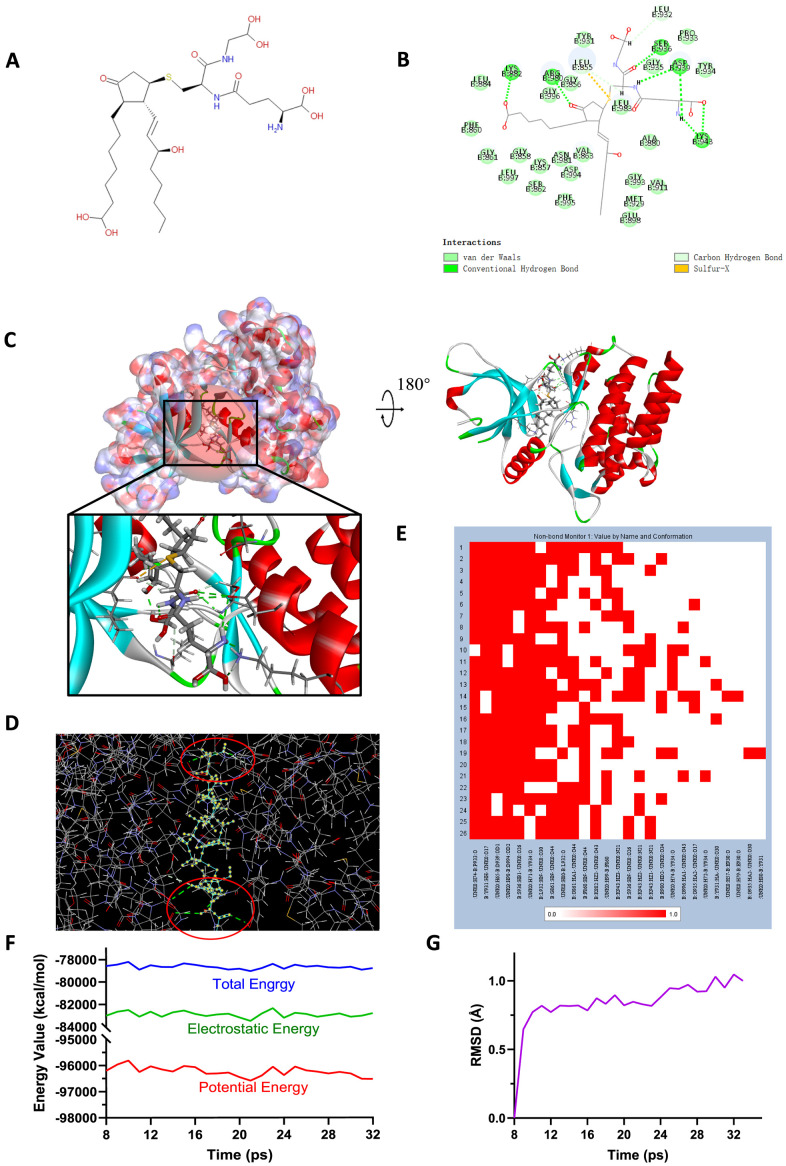
(**A**) Chemical structure of novel compound ZINC000004099068 selected from virtual screening. (**B**) Schematic drawing of inter-molecular interaction of the computed binding modes of ZINC000004099068 with JAK2. (**C**) Visualization of interactions between ligands and JAK2 (ZINC000004099068-JAK2 complex). The surface of binding area as well as active binding sphere were added. Blue represented positive charge, red represented negative charge and active binding sphere was shown as red region. Inhibitor was displayed in sticks, together with the structures around ligand-receptor junction were displayed in thinner sticks. (**D**) Mutual interactions between ZINC000004099068 and JAK2 under non-solvent environment after molecular dynamics simulation. The red circle showed chemical bonds existed in this complex. (**E**) Hydrogen bond heatmap in the progression of molecular dynamics. (**F**) Different kinds of energy values of ZINC000004099068-JAK2 complex. (**G**) Average backbone RMSD of ZINC00004099068-JAK2 complex. RMSD, root-mean-squared-deviation.

**Figure 7:**
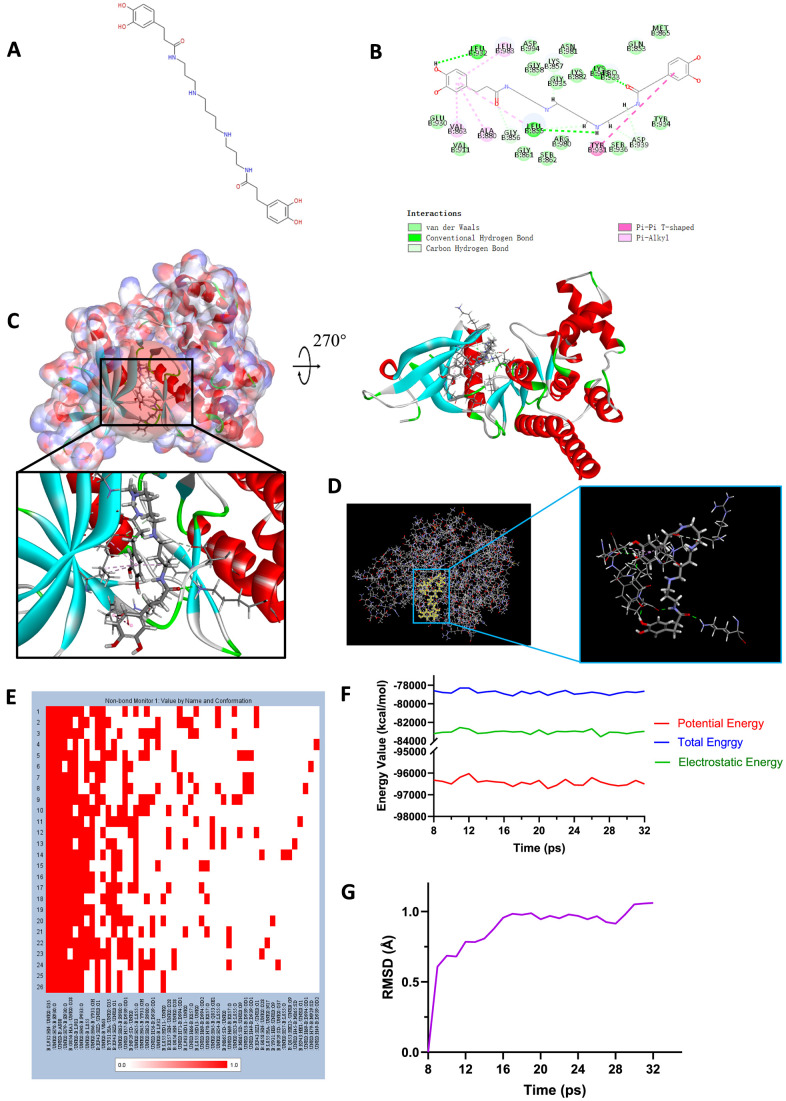
(**A**) Chemical structure of novel compound ZINC000013513540 selected from virtual screening. (**B**) Schematic drawing of inter-molecular interaction of the computed binding modes of ZINC000013513540 with JAK2. (**C**) Visualization of interactions between ligands and JAK2 (ZINC000013513540-JAK2 complex). The surface of binding area as well as active binding sphere were added. Blue represented positive charge, red represented negative charge and active binding sphere was shown as red region. Inhibitor was shown in sticks, together with the structures around ligand-receptor junction were shown in thinner sticks. (**D**) Mutual interactions between ZINC000013513540 and JAK2 under non-solvent environment after molecular dynamics simulation. Ligand was displayed in sticks and structures around ligand-receptor junction were displayed in thinner sticks. (**E**) Hydrogen bond heatmap in the progression of molecular dynamics. (**F**) Different kinds of energy values of ZINC00001351354-JAK2 complex. (**G**) Average backbone RMSD of ZINC000013513540-JAK2 complex. RMSD, root-mean-squared-deviation.

Safety should be taken into account when selecting candidate compounds. To predict the safety of the selected compounds, different kinds of indicators, including Ames mutagenicity, rodent carcinogenicity (based on the U.S National Toxicity Program (NTP) dataset) and developmental toxicity potential (DTP) properties were performed by TOPKAT module of DS4.5 (Table 6). Results depicted that 15 compounds were found to be non-mutagenic, 3 compounds were calculated to have non-rodent carcinogenicity and 2 compounds were discovered to have no DTP. As for the reference ligand Fedratinib, it was predicted to have non-carcinogenicity whether in rats or in mouse and no Ames mutagenicity, while it was discovered to have DTP property. Taking all the above-mentioned results into account, ZINC000013513540 and ZINC000004099068 were eventually selected as ideal lead compounds with non-CYP2D6 inhibition and non-hepatotoxicity, low rodent carcinogenicity, low ames mutagenicity, and less DTP compared to other compounds and Fedratinib. Consequently, ZINC000013513540 and ZINC000004099068 were identified as efficient, safe drug candidates and were pooled for subsequent research (Figures 6A, 7A).

**Table 6:** Toxicity predictions of candidate compounds.

**Number**	**Compounds**	**Mouse NTP**^1^		**Rat NTP**^2^		**AMES**^3^	**DTP**^4^
		**Female**	**Male**	**Female**	**Male**		
1	ZINC000013513540	0.139	0	0.02	0.84	0	1
2	ZINC000095620524	1	0	1	1	0	1
3	ZINC000056897657	0	0.98	1	0	1	1
4	ZINC000014951634	0.09	0	1	0	0	1
5	ZINC000011616635	0	1	1	1	1	1
6	ZINC000004096878	0	1	0	0	1	1
7	ZINC000014712793	0.64	0	1	0.63	0	1
8	ZINC000150338786	0	1	0	0	1	1
9	ZINC000085545908	1	0	0	0	0	1
10	ZINC000085544839	0	0.08	0	0.96	1	1
11	ZINC000062238222	0	0.07	0	0.96	0	1
12	ZINC000008552069	0.03	0	0	1	0.02	1
13	ZINC000004096684	0	1	1	0	1	1
14	ZINC000004099068	0	0	0	0	0	0.86
15	ZINC000004099069	0	0	0	0	0	0.86
16	ZINC000004654845	0	1	1	0	1	1
17	ZINC000096015174	1	0	1	0	0	0
18	ZINC000072131515	0	1	1	0	1	1
19	ZINC000085826837	0.20	1	1	1	0	1
20	ZINC000042805482	0.20	1	1	1	0	1
21	Fedratinib (reference)	0	0	0	0.04	0	0.90

^1^NTP: U.S National Toxicity Program. <0.15 (Non-Carcinogen); >0.85 (Carcinogen).

^2^<0.15 (Non-Mutagen); >0.85 (Mutagen).

^3^AMES: Ames mutagenicity. <0.15 (Non-Toxic); >0.85 (Toxic).

^4^DTP: Developmental toxicity potential.

### Molecular docking analysis and pesticide effect prediction

To study the ligand binding mechanisms of the two compounds and Fedratinib with JAK2, ZINC000013513540 and ZINC000004099068 were docked into JAK2 by CDOCKER module, which was a more accurate method than libdock for molecular docking. The RMSD between the initial docked posture and the ligand-JAK2 complex was calculated as 1.1219Å, proving that the CDOCKER module applied in this study was highly reliable for reproducing the experiment. Totally 10 conformations of each compound were generated and the CDOCKER interaction energy were calculated, the lowest energy of each compound was displayed in Table 7, the CDOCKER interaction energy of ZINC000013513540-JAK2 complex (-62.4674 kcal/mol) was significantly lower than the reference Fedratinib-JAK2 complex (-59.4672 kcal/mol), ZINC000004099068-JAK2 complex (-56.6172 kcal/mol) had the similar interaction energy as Fedratinib-JAK2 complex did, which elucidated these two compounds had the same or even higher binding affinity with JAK2 compared to Fedratinib. Hydrogen bonds and other chemical bonds were also applied to visualize the inter-molecule interaction between ligands and JAK2 (Figures 6B, 6C, 7B, 7C). Results showed that ZINC000013513540 formed 3 pairs of hydrogen bonds, 5 pairs of carbon hydrogen bonds, 1 pair of Pi-Pi interaction and 4 pairs of Pi-Alkyl bonds with JAK2. ZINC000004099068 formed 10 pairs of hydrogen bonds, 4 pairs of carbon hydrogen bonds, as well as 1 pair of sulfur bond with JAK2. As for the reference ligand Fedratinib, it formed 2 pairs of hydrogen bonds, 6 pairs of carbon hydrogen bonds, 1 pair of Pi-sigma bond, 3 pairs of alkyl bonds and 1 pair of Pi-alkyl bond with JAK2. The detailed information of chemical bonds was shown in Table 8. Additionally, this study further analyzed the pesticide effect of these two compounds: LD50 and LOAEL. As shown in Table 9, results indicated that ZINC000013513540 (LD50: 3.6 g/Kg, LOAEL: 107.2 mg/Kg) had relatively higher dosage of LD50 and LOAEL than the reference ligand Fedratinib (2.7 g/Kg, 347.0 μg/kg), ZINC000004099068 (LD50: 2.5 g/Kg, LOAEL: 1.4 g/Kg) had similar dosage of LD50 with Fedratinib and higher dosage of LOAEL than Fedratinib.

**Table 7:** CDOCKER interaction energy of compounds with Janus Kinase 2 (JAK2).

**Complex**	**CDOCKER interaction energy (Kcal/mol)**
ZINC000013513540-JAK2	-62.4674
ZINC000004099068-JAK2	-56.6172
Fedratinib-JAK2	-59.4672

**Table 8:** Chemical bond interaction parameters for each compound with JAK2 residues.

**Receptor**	**Compound**	**Interaction residues**	**Distances (Å)**	**Type**
JAK2	ZINC540	ZINC540:O1--LYS943:HZ3	2.53	Hydrogen Bond
		ZINC540:H55--LEU855:O	2.24	Hydrogen Bond
		ZINC540:H78--LEU932:O	1.89	Hydrogen Bond
		ZINC540:O28--GLY856:HA2	2.57	Carbon Hydrogen Bond
		ZINC540:H50-ASP939:OD1	2.67	Carbon Hydrogen Bond
		ZINC540:H53--ASP939:OD1	2.72	Carbon Hydrogen Bond
		ZINC540:H56--LEU855:O	2.98	Carbon Hydrogen Bond
		ZINC540:H62--LYS857:O	2.77	Carbon Hydrogen Bond
		ZINC540--TYR931	5.88	Pi-Pi interaction
		ZINC540--LEU855	4.31	Pi-Alkyl Bond
		ZINC540--VAL863	5.36	Pi-Alkyl Bond
		ZINC540--ALA880	5.38	Pi-Alkyl Bond
		ZINC540--LEU983	4.45	Pi-Alkyl Bond
	ZINC068	ZINC068:O43--LYS882:HZ1	1.76	Hydrogen Bond
		ZINC068:O26--SER936:HN	2.31	Hydrogen Bond
		ZINC068:O26--SER936:HG	3.06	Hydrogen Bond
		ZINC068:O23--LYS943:HZ2	2.07	Hydrogen Bond
		ZINC068:N21--LYS943:HZ3	1.86	Hydrogen Bond
		ZINC068:O34--ARG980:HH11	2.16	Hydrogen Bond
		ZINC068:H65--ASP939:OD1	2.48	Hydrogen Bond
		ZINC068:H71--ASP939:OD1	2.61	Hydrogen Bond
		ZINC068:H76--ZINC068:O17	3.06	Hydrogen Bond
		ZINC068:H81--ZINC068:O7	1.95	Hydrogen Bond
		ZINC068:O26--SER936:HB2	2.26	Carbon Hydrogen Bond
		ZINC068:O34--ARG980:HD1	2.45	Carbon Hydrogen Bond
		ZINC068:H64--LEU855:O	2.16	Carbon Hydrogen Bond
		ZINC068:H79--LEU932:O	2.86	Carbon Hydrogen Bond
		ZINC068:S12--LEU855:O	3.07	Sulfur Bond
	Fedratinib	4PS5:N18--LEU932:HN	2.08	Hydrogen Bond
		4PS5:H54--B:LEU932:O	1.96	Hydrogen Bond
		4PS5:O32--ASN981:HA	2.58	Carbon Hydrogen Bond
		4PS5:H38--4PS5:O8	2.63	Carbon Hydrogen Bond
		4PS5:H39--4PS5:O8	2.64	Carbon Hydrogen Bond
		4PS5:H48--B:LEU855:O	2.95	Carbon Hydrogen Bond
		4PS5:H49--B:LEU855:O	3.05	Carbon Hydrogen Bond
		4PS5:H55--B:GLU930:O	2.31	Carbon Hydrogen Bond
		4PS5--LEU855:HD23	2.60	Pi-Sigma Bond
		4PS5:C21--ALA880	3.58	Alkyl Bond
		4PS5:C21--B:VAL911	4.43	Alkyl Bond
		4PS5:C21--B:MET929	3.72	Alkyl Bond
		4PS5--B:ALA880	4.11	Pi-Alkyl Bond
		4PS5--B:LEU932	5.47	Pi-Alkyl Bond
		4PS5--B:LEU983	4.22	Pi-Alkyl Bond
		4PS5--B:VAL863	4.11	Pi-Alkyl Bond

ZINC540: ZINC000013513540; ZINC068: ZINC000004099068.

**Table 9:** Computation predictions of pesticide effect of two compounds and the reference ligand fedratinib.

**Compound**	**LD50**	**LOAEL**
ZINC000013513540	Computed Rat Oral LD50 = 3.6 g/kg	Computed Chronic LOAEL = 107.2 mg/kg
	Lower 95% Confidence Limits = 653.5 mg/kg	Lower 95% Confidence Limits = 3.5 mg/kg
	Upper 95% Confidence Limits = 10 g/kg	Upper 95% Confidence Limits = 3.3 g/kg
ZINC000004099068	Computed Rat Oral LD50 = 2.5 g/kg	Computed Chronic LOAEL = 1.4 g/kg
	Lower 95% Confidence Limits = 305.2 mg/kg	Lower 95% Confidence Limits = 30.4 mg/kg
	Upper 95% Confidence Limits = 10 g/kg	Upper 95% Confidence Limits = 10 g/kg
Reference ligand Fedratinib	Computed Rat Oral LD50 = 2.7 g/kg	Computed Chronic LOAEL = 347.0 μg/kg
	Lower 95% Confidence Limits = 409.9 mg/kg	Lower 95% Confidence Limits = 61.2 μg/kg
	Upper 95% Confidence Limits = 10 g/kg	Upper 95% Confidence Limits = 2.0 mg/kg

LD50: Lethal Dose, 50%.

LOAEL: Rat Chronic Oral Lowest Observed Adverse Effect Level.

### Molecular dynamics simulation

Molecular dynamics simulation was further conducted to test the stability of the ligand-JAK2 complex in the state of natural environment. The initial conformations were obtained from the molecular docking experiment through CDOCOER module. RMSD curves as well as energy values of these complexes were shown in Figures 6F, 6G, 7F, 7G. The RMSD trajectory of each complex (ZINC000004099068-JAK2, ZINC000013513540-JAK2) reached equilibrium after 16, 18 ps, respectively; energy values including potential energy, total energy and electrostatic energy of these complexes got stabilized with time. The results of molecular dynamics simulation visualized that these hydrogen bonds and other chemical bonds formed by compounds and JAK2 contributed the stability of these complexes (Figures 6D, 6E, 7D, 7E). Hydrogen bond heat map illustrated that the hydrogen bonds, which contributed largely to the stability of complex, could exist steadily with the progression of molecular dynamics.

## DISCUSSION

Primary Myelofibrosis (PMF) is a myeloproliferative neoplasm characterized by stem cell-derived clonal myeloproliferation, which is frequently but not always accompanied by gene mutation, abnormal cytokine expression, bone marrow fibrosis, anemia etc., [4, 17, 18]. PMF starts insidiously and progresses slowly, often with a long asymptomatic period before diagnosis, and patients could suffer from a series of symptoms [19–22]. Currently, the treatment of PMF primarily emphasizes on palliative treatment, aiming at relieving anemia, splenomegaly, constitutional symptoms, and bone pain. The reason caused this situation may be blamed for lacking effective diagnostic methods and biomarkers at the early stage of the disease [23, 24]. Accordingly, a comprehensive understanding of the molecular mechanism, progression as well as pathogenesis of PMF is imperative to formulate efficiently diagnostic and therapeutic strategies. Bioinformatics analysis combined with structural biology method was applied in this study to fully investigate the PMF from the carcinogenesis to the treatment, and assess the properties of existed drug Fedratinib in the meantime.

In the current study, 54 PMF samples and 72 normal samples were extracted from 3 mRNA microarray series from GEO. Firstly, raw data (“.CEL” format) was downloaded for bioinformatics analysis. Then this study preprocessed the raw data as well as discussing the reason for data preprocessing: GEO do not take responsibility for uploaders’ chips quality, so it remains unknown what algorithm the author used in their “series_matrix.txt” file, different “series_matrix.txt” file might be generated from different algorithms, platforms or time. Some matrices’ gene expression levels may fluctuate at ten whereas others approach tens of thousands, which invisibly increases the difficulty to compare more than one dataset for study. The purpose of data standardization was to eliminate high co-relevance generated by systematic bias while preserving high correlation of gene expression level caused by the real biological reason. Through RMA algorithm, standard gene expression profiles data were obtained by background correction and standardized pretreatment. After that, RNA degradation image of each GSE series as well as NUSE box plot was plotted to verify the chips’ quality, and 2 samples were removed due to their poor chip quality. Next, “batch effects” were eliminated for the gene expression profiles, which was due to sample preparation or array variation (charge, type, and/or platform), it could mask or confound the real difference among biology [15, 16, 25]. Non-biological experimental variation or “batch effects” was commonly observed across multiple batches of microarray experiments, which rendered the task of combing data from different batches difficult. In order to check batch effects among these 3 series, principal component analysis (PCA) was performed, and PCA image was plotted, results illustrated that these 3 datasets were clustered in different places, which meant they had a different pattern of gene expression. PCA analysis was conducted again after batch effects were removed, and results displayed that the points of 3 datasets interleaved with each other, with almost no outliers. PCA analysis provided solid evidence for either the existence or elimination of batch effects. Consequently, eliminating batch effects was essential for researchers to analyze the real difference in the gene expression profiles.

Next, bioinformatics analysis was conducted to find out the hub genes of PMF. A total of 810 DEGs, 337 DEGs and 1742 DEGs were identified from 3 datasets, respectively. Altogether, 95 mutual DEGs were obtained among those 3 datasets, as shown in Venn plot. After GO analysis of abnormal expression genes, we detected that those up-regulated genes were mainly associated with erythrocyte differentiation, killing of cells of other organism, ATP binding, protein binding and hemoglobin complex, which explained why the fast multiplication of tumor cells as well as the destroy of normal cells. Down-regulated genes were primarily involved in humoral immune response, transcription factor binding and plasm membrane. Our results suggested that the abnormal changes including erythrocyte differentiation, neutrophil degranulation may occur in hemoglobin complex, which agreed with the previous study that obstacle to myeloproliferative differentiation may cause aplastic anemia in the progression of PMF [26]. Some studies have also demonstrated that cell membrane ion channels, such as iron ion binding, participate in cell signal transduction, proliferation, apoptosis as well as regulation of gene expression in tumor levels [27, 28]. Furthermore, analyses of KEGG and GSEA revealed that the mutual up-regulated DEGs were chiefly enriched in the cell cycle, JAK-STAT signaling pathway and TNF signaling pathway. Previous studies have reported that tumor necrosis factor (TNF) interacts with tumor cells to trigger cytolysis or cell death. TNF could also promote inflammatory responses, which acts via the TNF receptor (TNFR) and is part of the extrinsic pathway for triggering apoptosis [29–31]. Advanced studies have further reported that dysregulation of the cell cycle accelerated the carcinogenesis of tumor [32–34], and drugs acting on the cell cycle may benefit patients [35], which was subscribed to our findings that cell cycle was aberrantly activated in PMF. JAK-STAT signaling pathway would be discussed in detail later.

With the aim of screening hub genes among DEGs identified in our former work, the 95 mutual DEGs were analyzed with construction of PPI network based on the STRING database. 11 different algorithms were applied in Cytoscape software (“cytohubba” plug-in), the final hub genes were selected by intersecting the top 25 genes in each algorithm, which could provide a solid reliability of the selected hub genes, including LCN2, JAK2, MMP8, CAMP, DEFA4, LTF, MPO, HBD, STAT4, EBF1. Particularly JAK2 and LCN2 ranked prominent in these 11 algorithms, suggesting that they were the most essential genes in the occurrence of PMF.

LCN2, located on chromosome 9q34, is a secreted protein that belongs to the lipocalins, a group of transporters of small lipophilic molecules such as iron, steroids, fatty acids and lipopolysaccharides in circulation [36]. The altered expression of LCN2 could trigger disease in several pathologic organs, including liver injury, steatosis, kidney injury, brain injury, cardiomyopathy, musculoskeletal disease and cancer of several organs [37]. Previous research cloned the full length of human LCN2 cDNA and results demonstrated that this lipocalin was mainly expressed in myeloid cells [38]. Existed studies accessed LCN2 protein levels in the serum between PMF samples and healthy donors [39], results indicated that the level of LCN2 secreted protein was significantly higher in PMF patients than in healthy donors, suggesting that LCN2 could be considered as a biomarker. In the course of last decades, LCN2 had been well studied as a potential biomarker whether for the kidney injury or to estimate the outcome of different diseases. In one multicenter, prospective cohort study, a significant correlation between urinary injury biomarkers (LCN2, KIM-1, IL-8, L-FABP and albuminuria) and the disease outcome indicated that LCN2 only distinguished progression alone from non-progression, elucidating that LCN2 could be potentially served to recognize patients who were at high risk of progression [40]. Those studies provided solid evidence for the utility of LCN2 as a biomarker, and LCN2 could also be a promising diagnostic and therapeutic target of PMF in our study.

Until now, four JAKs have been recognized in mammals: JAK1, JAK2, JAK3 and TYK2, of which Janus Kinase 2 (JAK2) is a cytoplasmic tyrosine kinase engaged with numerous signaling pathways, referring interleukin-3, granulocyte colony-stimulating factor, granulocyte-macrophage colony-stimulating factor, receptors for erythropoietin and thrombopoietin [41]. The pathogenetic contribution of JAK2 is currently thought to involve the upregulation of JAK-STAT signaling pathway [42]. JAK-STAT signaling pathway is a signal transduction pathway stimulated by cytokines, which participated in many necessary biological processes, such as cell proliferation, differentiation, apoptosis, and immune regulation. The mechanism of JAK signal transmission is that binding of cytokines to the corresponding receptors leads to the dimerization of receptor molecules, making the receptor-coupled JAK kinases close to each other and activated by Interactive tyrosine phosphorylation. After activation of JAK, the tyrosine residues on the receptor are phosphorylated, and then these phosphorylated tyrosine sites form a “berthing site” (docking site) with the surrounding amino acid sequence, which also contains the SH2 domain recruited to this docking site. Finally the STAT protein which bound to the receptor is phosphorylated by kinase JAK, and the activated STAT protein enters in nucleus in the form of dimer to bind to the target gene and regulates gene transcription [43–45]. None of references pointed that JAK2 had any pivotal cellular activity to maintain the functions of the body, which elucidated the specificity of JAK2. Therefore, JAK2 is an indispensable therapeutic target without participating in any other pivotal cellular activity, which could be inhibited to prevent the JAK-STAT signaling pathway and thus prevent the occurrence of PMF.

At present, there is no effective treatment for PMF, namely there is no radical way to cure this disease [6]. Based on the anomalous activation of JAK-STAT signaling pathway in PMF, several studies have reported their discovery of JAK2 inhibitors [46, 47]. Unfortunately, current drug therapy in PMF including JAK2 inhibitor lacks disease-modifying activity. Despite the fact that great progress with inhibitors has been made regarding JAK2 in medication design and development, merely Fedratinib, which was selected as the reference drug in this study, has displayed a relatively mature research until now [9].

Though Fedratinib, which is currently being tested as a potential anti-cancer compound, has therapeutic potential in anti-neoplastic fields, it still had great therapeutic limitations. Relevant research showed that common adverse events with Fedratinib treatment were anemia, gastrointestinal symptoms, increased levels of serum creatinine, liver transaminases as well as pancreatic enzymes. Besides, Wernicke encephalopathy of unknown reason were found when treatment with Fedratinib [9]. Consequently, discovering more compounds targeting JAK2 is of profound significance for pharmacology as well as clinical application. Based on the previous outcomes in this study, structural biology was performed to screen natural inhibitors targeting JAK2 for further investigation.

In structural biology study, 17799 biogenic-for sale-named compounds were obtained from ZINC15 database for virtual screening, followed by ADME, TOPKAT, CDOCKER and Molecular Dynamics Simulation. Libdock module was firstly conducted in order to screen appropriate compounds which could dock with JAK2 through a fast docking analysis from the tremendous ligands. Libdock score represents the energy optimization and stability of the conformation. Compounds with a higher libdock score indicates a better energy optimization and a more stable conformation. Altogether, 10102 compounds were found eligible to bind with JAK2 stably after libdock module. Among those, 667 compounds were calculated to have higher libdock score than the reference ligand Fedratinib (libdock score: 129.056), suggesting that these 667 compounds may form a more stable conformation together with a better energy optimization compared to Fedratinib. Based on libdock score, the top 20 natural compounds were selected and pooled for following study.

Pharmacologic properties including ADME and toxicity were predicted to access these selected compounds. Taking all the properties as well as safeties into account, two compounds, ZINC000013513540 and ZINC000004099068, were identified as ideal lead compounds. Their properties of being soluble in water and easily absorbed by the intestine could alleviate the gastrointestinal symptoms compared to Fedratinib. Additionally, Fedratinib was predicted with hepatotoxicity while these two compounds were non-hepatotoxicity, which could reduce the damage to the liver. As for CYP2D6, cytochrome P450 (CYP450) is the principal enzyme involved in drug metabolism. Drugs behaved as inhibitors of CYP450 could weaken the activity of drug enzymes and thus slow down the drug metabolism. CYP2D6 is one of the enzymes in CYP450, and these compounds were predicted with non-CYP2D6 inhibition, suggesting that these two compounds could make the drug enzyme function greatest in drug metabolism. Furthermore, they had weak plasma protein binding property, the free part of ligand could behave the best pesticide effect, which demonstrated the good selectivity of the compounds regarding JAK2: compounds won’t be bound by other substances in the body such as plasma protein and thus reduced the combination with JAK2. However, only this molecule was analyzed, which was inadequacies in this study, it still remained unknown whether the drugs could be combined by other substances in the body, since different selectivity of drugs resulted in different dosage in future vitro/vivo experiment. Further study ought to be focused on the selectivity of the inhibitor. Moreover, these two compounds were accessed with non-Ames mutagenicity, low rodent carcinogenicity and less developmental toxicity potential compared to other compounds, which strongly suggested their perspective application in drug development. Although the remaining compounds on the list (Tables 5, 6) possessed relatively negative effects and toxicity, they still had potential application for the drug design and refinement by adding or removing specific groups and atoms to obtain better pharmacologic properties. Thus, ZINC000013513540 and ZINC000004099068 were finally selected as ideal lead compounds and were applied in further analysis.

Docking mechanism as well as chemical bond interaction of candidate compounds with JAK2 was then analyzed and visualized. CDOCKER module computation results demonstrated that these two compounds had the same or even higher binding affinity with JAK2 compared to Fedratinib. Subsequently, the chemical structures of the two compounds as well as Fedratinib with JAK2 were conducted with intuitive visualization (Figures 6, 7). The two compounds combined with JAK2 had more chemical bonds than Fedratinib did, which could maintain a more solid structure and thus prevent the function of JAK2 by contributing a competitive inhibition. In addition, results of pesticide effect (LD50 and LOAEL) of these two compounds indicated that they were relatively safe drugs as Fedratinib did, that they won’t have rapid adverse reactions due to high dosage of these drugs. Meanwhile, their properties of non-inhibition with liver drug enzyme CYP2D6 also proved the safety of these compounds. Consequently, the computational predictions could provide a reference for future experimental verification.

Lastly, molecular dynamics simulation was performed to verify their stability under natural circumstances. RMSD and energy values were determined as evaluation indexes. Results revealed that the RMSD trajectories of complexes reached equilibrium after 16, 18 ps, respectively. Energy curves got stabilized with time, hydrogen bond heat map illustrated that the hydrogen bonds, which contributed largely to the stability of complex, could exist stably with the progression of molecular dynamics. In conclusion, these two compounds, ZINC000013513540 and ZINC000004099068, could interact with JAK2 stably, and their complexes could exist under natural environment steadily, as well as inhibiting the function of JAK2 just the same as Fedratinib did.

Based on all the results above, drug development, including modification and refinement, could be prospectively conducted to make ligands and receptors bind more stably. It is also noteworthy that agonists and inhibitors always share a similar skeleton in the chemical structure [10, 11], adding or removing different groups or atoms may cause the opposite effects. Combining the advantages of pharmacologic properties, highly binding affinity as well as stabilization with JAK2 of these two compounds, they could provide a valuable resource for JAK2-related medication development.

**Figure 8:**
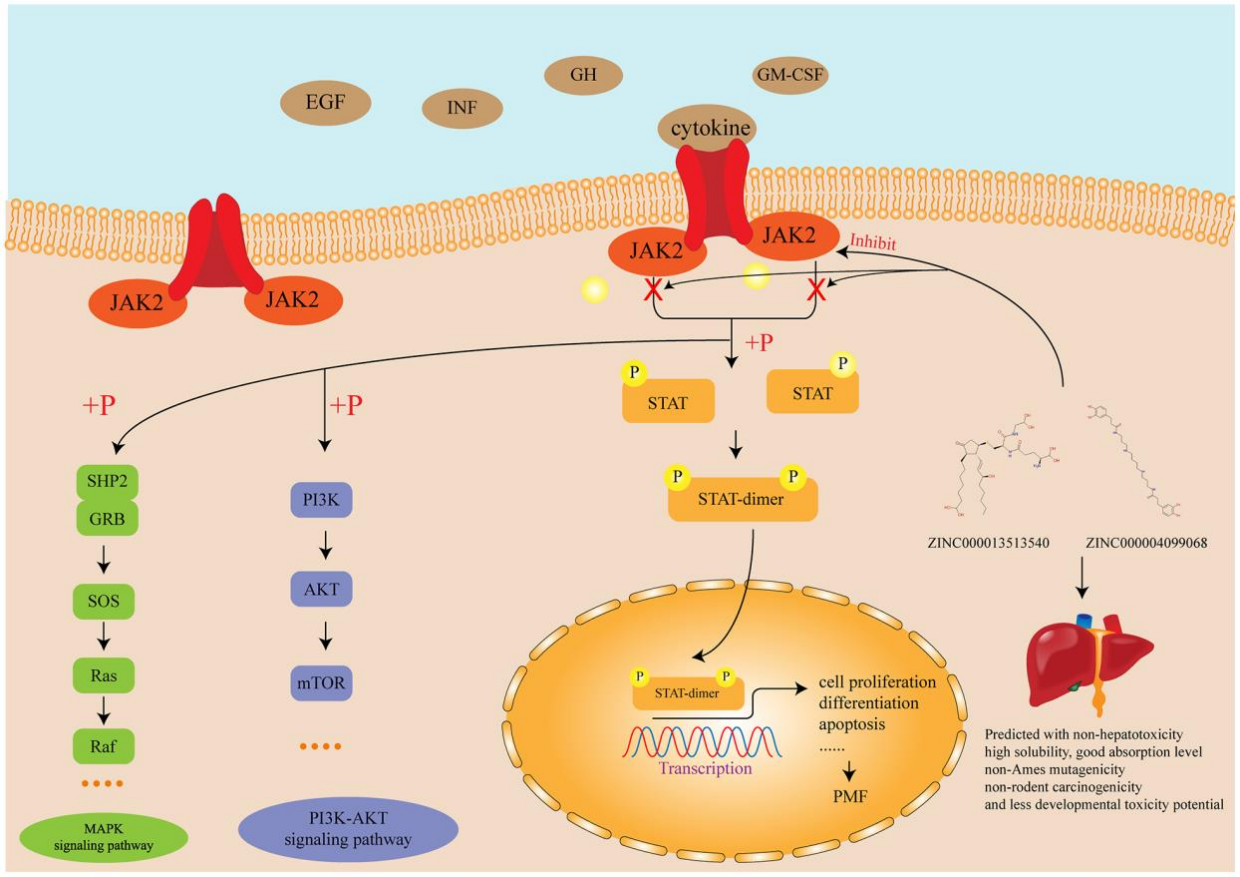
**Diagram of the JAK-STAT signaling pathway and other related signaling pathway as well as the mechanism of the function of inhibitors identified in this study.**

Overall, in this study, from the identification of DEGs by bioinformatics to the study of inhibitors by structural biology, each step had been adequately explained. This study provided a guideline for preprocessing the GSE series’ data, as well as screening lead compounds which could have potential effects regarding receptor. The JAK-STAT signaling pathway and other relevant signaling pathway and the mechanism of the function of inhibitors identified in this study were illustrated in Figure 8. Firstly, we fully discussed the selection, downloading and preprocessing of GSE series’ data. Through a series of analytical methods, ten genes (LCN2, JAK2, MMP8, CAMP, DEFA4, LTF, MPO, HBD, STAT4, EBF1) were identified as hub genes responsible for the carcinogenesis of PMF. Of which, two genes, JAK2 and LCN2 were the most essential genes and fully discussed. Next, structural biology was performed to screen natural compound inhibitors regarding JAK2 based on the meager research status. Molecular conformation, pharmacologic properties, binding affinity as well as stability of each candidate compounds were fully investigated to verify their superiority compared to Fedratinib. ZINC000013513540 and ZINC000004099068, were finally selected as the ideal lead compounds, which may have potential effect in current chemotherapy of PMF. It is also worth noting that there is no single drug that could be marketed directly, unless through thousands of refinements, improvements, and finally clinical tests. Based on the two compounds selected in this study, further research could focus directly on the improvement and modification of them.

Although this study was conducted by elaborate design and precise methods have been carried out, it still had some limitations. Further experiments including animal test and cell experiment, were required to verify our results more firmly. Besides, more substances, like the plasma protein analyzed in this study, should be assessed in further studies in order to test whether these compounds were effectively selective inhibitor of JAK2.

## CONCLUSIONS

This study combined two parts of bioinformatics and structural biology. First, after analysis of bioinformatics, this study found out two significant hub genes: JAK2 and LCN2, which were remarkably highly expressed and were responsible for the progression of PMF. Next, based on the JAK2, structural biology method was conducted, after a series of computer-aided structural and chemistry techniques (e.g., virtual screening, molecular docking, pharmacologic properties prediction, molecular dynamics simulation), two compounds, ZINC000013513540 and ZINC000004099068, were selected as safe and effective drug candidates, they were promising drugs in the treatment of patients with PMF, which could also contribute greatly to JAK2 inhibitors’ development and refinement.

## MATERIALS AND METHODS

### Microarray data

Gene expression profiles of GSE26049, GSE53482, GSE61629 were downloaded from the GEO database (https://www.ncbi.nlm.nih.gov/geo/), which is a functional public genomics dataset including high throughput gene expression data, chips and microarray. Multiple sample sets were utilized in this study to avoid clinical bias and race among different studies. Totally 55 PMF samples and 73 normal samples were provided on platforms “GPL570, Affymetrix Human Genome U133 Plus 2.0 Array” (GSE26049, GSE61629) and “GPL13667, Affymetrix Human Genome U219 Array” (GSE53482). Of which, GSE26049 contained 9 PMF samples and 21 normal samples, GSE53482 included 42 PMF samples and 31 normal samples and GSE61629 provided 4 PMF samples and 21 control samples.

### Gene expression profiles’ preprocessing

This study used raw data (“.CEL” file format) from each GSE profiles in that GEO website took no responsibility for uploaders’ chips quality control. Firstly, the gene chips’ quality control was performed by R language (“affy”, “affyPLM” package), standard samples involved in this study were identified through calculating their normalized unscaled standard errors (NUSE), plotting RNA degradation figure, grayscale image, residual figure etc. After identifying standard samples, we conducted “RMA” algorithm (“rma” function in R) to perform background correction and standardized pretreatment on the gene expression profile data to obtain the standard gene expression data. Lastly, “batch effects” were removed among these gene profiles through “combat” function in R (“sva” package).

### Identification of DEGs

The differentially expressed genes (DEGs) between PMF and normal samples were screened using R (“limma” package, the most widely used package in Bioconductor repository to analyze DEGs). “limma” package allows researchers to compare two or more datasets in GSE series in order to discover DEGs across experimental conditions. The false discovery rate (Benjamini-Hochberg algorithm) and adjusted P-values were applied to provide a balance between discovery of statistically significant genes and limitations of false-positives. Probe sets without corresponding genes or multiple probe sets corresponding to one gene were removed or averaged in the gene expression value, respectively. Each 2 groups were compared to identify DEGs between PMF and normal samples, the DEGs were calculated by adjusted P-value with cutoff of < 0.05 and |logFC| (Fold Change) >1, which was considered to be statistically significant. Subsequently, Venn plot analysis regarding each DEGs was applied among up-regulated, down-regulated, and total DEGs (“VennDiagram” package in R).

### Functional and pathway enrichment analysis on DEGs

DAVID database (Database for Annotation, Visualization and Integrated Discovery, http://david.abcc.ncifcrf.gov/) is a biological essential online repository that provides a comprehensive set of functional annotation tools for researchers to extract biological information underlying different genes. GO (Gene Ontology) is a major bioinformatic tool to analyze biological process, molecular function and cellular component among different genes. KEGG (Kyoto Encyclopedia of Genes and Genomes) is a resource database for understanding diverse signaling pathways and genomic information links. GO and KEGG analyses were performed in DAVID database for identified DEGs, P < 0.05 was set as the threshold as statistically significant definition. Gene sets with statistical significance were further determined by Gene Set Enrichment Analysis (GSEA).

### Protein-protein interaction network construction and module selection

The PPI network analysis was performed on STRING (Search Tool for the Retrieval of Interacting Genes, https://string-db.org/), an online database which was applied to visualize the connection between different genes. Then, Cytoscape software (version 3.7.0, an open source bioinformatic software platform for visualizing molecular interaction networks) was conducted to screen hub genes and modules among mutual DEGs through “cytohubba”, “MCODE”, respectively. Molecular Complex Detection (MCODE) is a plug-in for clustering given network links based on topology to find densely connected regions. “Cytohubba” is another plug-in which provides 11 different topological methods including “MCC”, “DMNC”, “MNC”, “Degree”, “EPC”, “BottleNeck”, “EcCentricity”, “Closeness”, “Radiality”, “Betweenness”, “Stress”, aiming at identifying key targets and sub-network from a complicated network. The significant modules were screened by “MCODE” while hub genes were identified by “ctyohubba”. The hub genes were obtained by calculating score then aligning the top 25 genes of each method and finally taking the intersection of these genes. Next, GO and KEGG analyses of significant modules were performed again using DAVID database.

### Docking software and ligand library

This study used Discovery Studio (version 4.5, BIOVIA, San Diego, California, USA) for further investigation, which is a suite software for simulating small molecules and macromolecules system. Discovery Studio (DS) is developed aiming at screening, designing and modifying potential drugs by applying structural chemical and structural biologic computation. Large amount of lead compounds as well as drug candidates have been identified through this method. Libdock module in DS was employed for virtual screening; CDOCKER module was used for docking analysis; ADME and TOPKAT modules (absorption, distribution, metabolism, excretion, and toxicity prediction) were analyzed for pharmacologic properties. The natural lead compounds obtained in this study was from ZINC15 database, a natural product repository for development and research of compounds. It was a free repository of commercially available compounds provided by the Irwin and Shoichet Laboratories among department of Pharmaceutical Chemistry, University of California, San Francisco.

### Structure-based virtual screening using libdock

**Figure 9:**
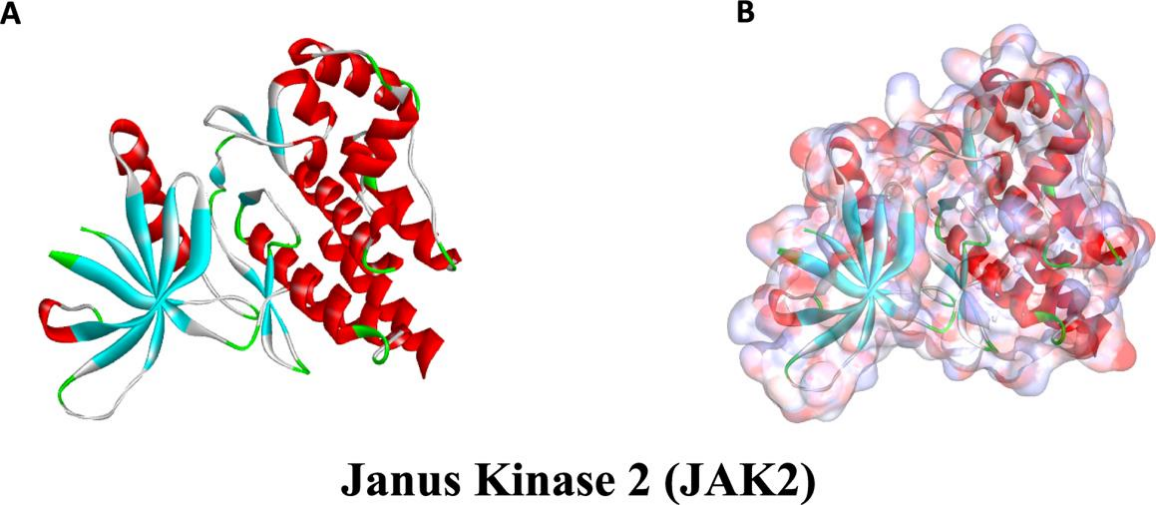
**The molecular structural of Janus Kinase 2 (JAK2).** (**A**), Initial molecular structure. (**B**), Surface of binding region added. Blue represented positive charge and red represented negative charge.

Ligand binding pocket region of Janus Kinase 2 (JAK2) was selected as the binding site to screen compounds which could potentially dock at to inhibit JAK2. Virtual screening was carried out using libdock module of DS4.5 [48]. Libdock (San Diego, CA, USA) is a rigid-based docking program, which calculates hotspots for protein using a grid placed into the binding site and polar and apolar probes. Next, the hotspots are further used to align the ligands to form favorable interactions. The Smart Minister algorithm and CHARMm force field (Cambridge, MA, USA) were performed for ligands minimization. After minimization, all the ligand postures were ranked based on ligands score. The 2.40 Å crystal structure of JAK2 (PDB ID: 4JI9) and its inhibitor Fedratinib (TG101348, PDB ID: 4PS5) were downloaded from RCSB Protein Data Bank (PDB) and imported into system working environment of libdock. The crystal structure of JAK2 was illustrated in Figure 9. JAK2 protein was prepared for docking by removing crystal water and other hetero atoms surrounding it, so that the negative effects of fixed water molecules could be eliminated, followed by addition of hydrogen, ionization and protonation. The CHARMm force field and the Smart Minimiser algorithm were employed for energy minimization [49]. The active site for docking was generated by extracting the initial ligand docked with JAK2 and then the binding site was defined from “edit binding site” option on the receptor-ligand interaction tool bar. Virtual screening was then carried out by docking all the prepared ligands obtained from ZINC15 repository at the defined active binding site. All the docked postures were ranked and grouped based on libdock score.

### ADME and TOPKAT predictions

ADME (absorption, distribution, metabolism and excretion) module in DS4.5 was employed to calculate ADME pharmacologic properties of selected compounds, including their aqueous solubility, blood-brain barrier penetration, cytochrome P450 2D6 (CYP2D6) inhibition, hepatotoxicity, plasma protein binding level and human intestinal absorption. TOPKAT (Toxicity Prediction by Komputer Assisted Technology) module of DS4.5 was also employed to calculate the toxicity and other properties of potential compounds, such as Ames mutagenicity, development toxicity potential (DTP), rodent carcinogenicity etc. These pharmaceutical properties of compounds were fully considered when selecting drug candidates for JAK2.

### Molecular docking visualization and pesticide effect prediction

CDOCKER module of DS4.5 was conducted for molecular docking study. CDOCKER is a precise molecular docking method for ligands and receptors based on the CHARMm force field, which could provide high-precision results for analysis. Receptor is held rigid while ligands are allowed to flex during the docking process. For each complex posture, the interaction energy, which indicated the ligand binding affinity, was calculated. Crystal structure of JAK2 was obtained from PDB, and crystal water molecules were generally removed in a rigid and semi-flexible docking process in that the fixed water molecules might affect the formation of receptor-ligand complex [50]. Subsequently, hydrogen atoms were added to the protein. To test the reliability of the combination mode of this system, the initial ligand of JAK2 was extracted from the binding site and then re-docked into the receptor in order to calculate the root-mean-squared-deviation (RMSD) between these two conformations.

The binding site sphere of JAK2 was defined as the region which came within 5 Å radius from the geometric centroid of the initial ligand. Structures of identified hits were prepared and docked into the binding pocket of JAK2. The radius of the binding cavity was set as 13 Å to allow diverse ligands to dock. Top hits of ligands were set as 10, and pose cluster radius was set as 0.5 to generate more ligand conformations, allowing ligands flex enough to obtain the most fitted conformation as well as the lowest energy values with JAK2. Different postures of each ligand-receptor complex were generated, and interactions within complex were visualized in DS4.5. The cluster ranking was performed based on the CDOCKER interaction energy representative of each cluster. Complexes with the best posture together with suitable interaction energy could be chosen for further investigation. Next, computational predictions of pesticide effect of the chosen compounds were carried out to assess the pharmacologic properties as well as providing reference for future experimental verification.

### Molecular dynamics simulation

The stability of the best binding conformations of the complexes chosen among those poses was further validated by molecular dynamics simulation. The ligand-receptor complex was put into an orthorhombic box and solvated with an explicit periodic boundary solvation water model. Sodium chloride was added to the system with the ionic strength of 0.145 in order to simulate the physiological environment in body. Then, the system was subjected to the CHARMm force field and relaxed by energy minimization (500 steps of steepest descent and 500 steps of conjugated gradient), with the final root-mean-square (RMS) gradient of 0.305. The entire system was driven slowly from initial temperature of 50K to the target temperature of 300K for 2 ps, and equilibration simulation run for 5 ps. The production module of molecular dynamics simulation run for 30ps with time step of 1 fs. The simulation was employed with the NPT (normal pressure and temperature) system at a constant temperature of nearly 300 K during the whole process. The particle mesh Ewald algorithm was used to calculate long-range electrostatics, and the linear constraint solver algorithm was adapted to fixing all bonds involving hydrogen. With the initial ligand-receptor complex setting as reference, a trajectory was determined for RMSD, potential energy, inter-molecule interaction, and structural characteristics, respectively, through the DS4.5 trajectory protocol analysis.

### Data availability statement

The data used and analyzed during the current study are available upon reasonable request.

### Consent for publication

All contributing authors agree to the publication of this article.

## Supplementary Material

Supplementary Figures
