# Supplementary material for: Transcriptome profiling reveals target in primary myelofibrosis together with structural biology study on novel natural inhibitors regarding JAK2
Source: Aging (Albany NY). 2021 Mar 3;13(6):8248–75. doi: 10.18632/aging.202635 (PMC8034969; doi:10.18632/aging.202635)
Supplement: Supplementary Figures [file aging-13-202635-s001.pdf]

SUPPLEMENTARY FIGURES

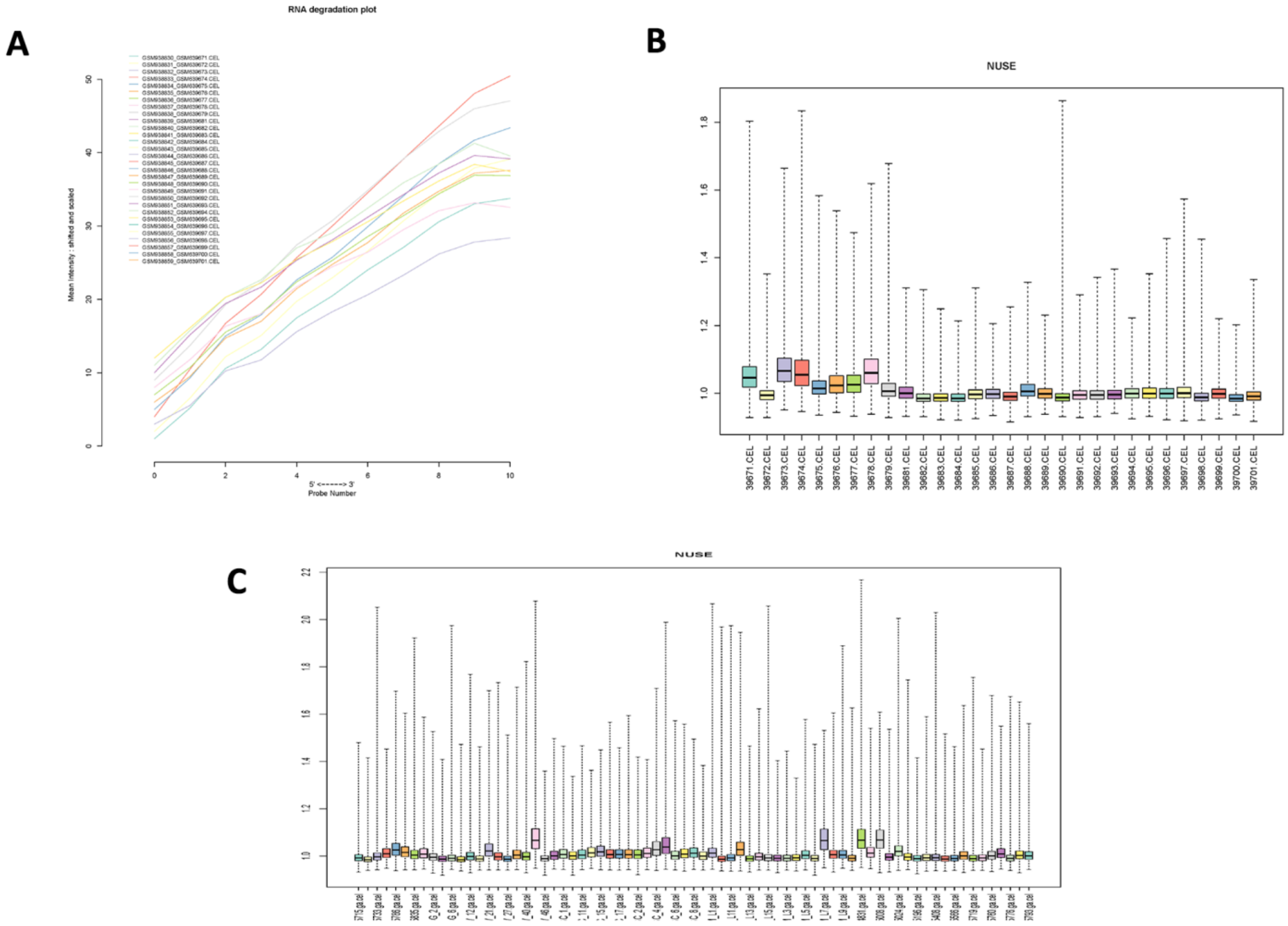

Supplementary Figure 1. (**A**), RNA degradation plot of GSE26049. (**B**), Box plot of normalized unscaled standard errors (NUSE) of GSE26049 and (**C**), Box plot of normalized unscaled standard errors (NUSE) of GSE53482, which were used for quality control.

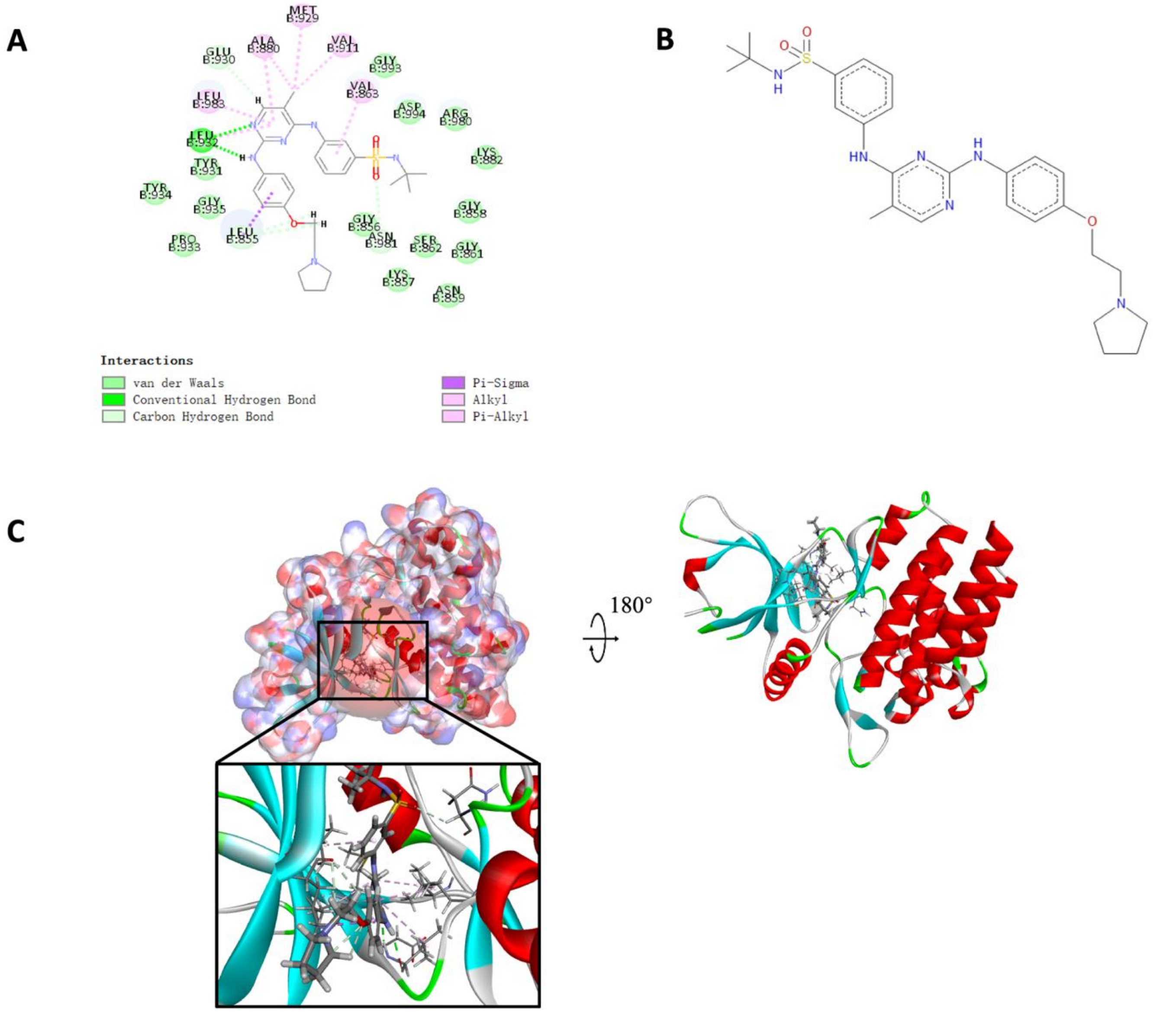

Supplementary Figure 2. (**A**), Schematic drawing of inter-molecular interaction of the computed binding modes of Fedratinib with JAK2. (**B**), Chemical structure of compound Fedratinib selected as reference drug in this study. (**C**), Visualization of interactions between ligands and JAK2 (Fedratinib -JAK2 complex). The surface of binding area as well as active binding sphere were added. Blue represented positive charge, red represented negative charge and active binding sphere was shown as red region. Reference inhibitor Fedratinib was displayed in sticks, together with the structures around ligand-receptor junction were displayed in thinner sticks.
